# Interaction of sleep duration and depression on cardiovascular disease: a retrospective cohort study

**DOI:** 10.1186/s12889-022-14143-3

**Published:** 2022-09-15

**Authors:** Bowen Jin, Hang Zhang, Fuchun Song, Guangjun Wu, Hui Yang

**Affiliations:** 1 Department of Cardiac Surgery, Wuhan Asia Heart Hospital, Wuhan, 430022 Hubei P.R. China; 2 Beijing Union University, Beijing, 100101 P.R. China; 3 Immunology Laboratory, Guang’anmen Hospital, China Academy of Chinese Medical Sciences, No.5 Beixiange, Xicheng District, Beijing, 100053 P.R. China

**Keywords:** CHARLS, Sleep duration, Depression, CVD, Interaction

## Abstract

**Background:**

To assess the interaction of sleep duration and depression on the risk of cardiovascular disease (CVD).

**Methods:**

A total of 13,488 eligible participants were enrolled in this retrospective cohort study eventually. Baseline characteristics were extracted from the China Health and Retirement Longitudinal Study (CHARLS) database, including age, sex, diabetes, high-density lipoprotein (HDL), blood glucose (GLU), glycosylated hemoglobin (GHB) etc. Univariate and multivariate negative binomial regression models were carried out to assess the statistical correlation of sleep duration and depression on CVD separately. Additionally, multivariate negative binomial regression model was used to estimate the interaction of sleep duration and depression on CVD risk.

**Results:**

After adjusting for age, sex, educational background, hypertension, diabetes, dyslipidemia, the use of hypnotics, disability, nap, drinking, deposit, sleep disturbance, HDL, triglyceride, total cholesterol, GLU and GHB, the risk of CVD in participants with the short sleep duration was increased in comparison with the normal sleep duration [relative risk (RR)=1.02, 95% confidence interval (CI):1.01-1.03]; compared to the participants with non-depression, participants suffered from depression had an increased risk of CVD (RR=1.05, 95%CI:1.04-1.06). Additionally, the result also suggested that the interaction between short sleep duration and depression on the risk of CVD was statistically significant in these patients with diabetes and was a multiplicative interaction.

**Conclusion:**

An interaction between short sleep duration and depression in relation to an increased risk of CVD among Chinese middle-aged and elderly individuals was noticed, which may provide a reference that people with diabetes should focus on their sleep duration and the occurrence of depression, and coexisting short sleep duration and depression may expose them to a higher risk of CVD.

**Supplementary Information:**

The online version contains supplementary material available at 10.1186/s12889-022-14143-3.

## Background

Cardiovascular disease (CVD), as a common chronic disease, is still the main cause of global mortality and disability [1, 2]. In recent years, the incidence of CVD has remained a steady rise globally, reaching 523 million in 2019 [2]. It is estimated that there was 17.9 million people died of CVD every year, accounting for 32% of global deaths [3], brought huge burden of disease for many families. As a consequence, it is very important for closely paying attention to risk factors to prevent the occurrence of CVD.

To date, several studies have reported that poor behavior and mental health are closely associated with the CVD risk, including short sleep duration, long sleep duration, and depression [4, 5]. A systematic review and meta-analysis showed that both short and long sleep duration were markers of cardiovascular outcomes, and were also associated with a higher risk of coronary heart disease (CHD) [6]. In addition, Yin, et al. also pointed out that there was a U‐shaped association between sleep duration and risk of CVD, and insufficient or excessive sleep duration were significantly related to an elevated risk of CVD [7]. Depression as a mental illness of low mood and loss of interest, has become increasingly common worldwide [8]. A growing body of scientific evidence have evaluated the role of depression in the development of CVD [8, 9]. In the study of Carney, et al., the result showed that depression was recognized as a highly prevalent risk factor for CHD occurrence [10]. The association mechanism of depression and CVD risk might be associated with the vascular endothelial dysfunction and increased platelet aggregation among patients with depression, thus accelerating the development of CVD [11]. Notably, there were some studies have showed a bidirectional relationship of sleep duration and depression [12, 13]; insufficient or excessive sleep duration could increase the risk of depression [12]. Simultaneously, people with depression could bring a short sleep duration [13]. Although short/long sleep duration and depression have been considered as risk factors for the development of CVD, people with combined short/long sleep duration and depression may represent a population with a higher risk of CVD due to a possible interaction of short/long sleep duration and depression. There were few studies, to our knowledge, have assessed the influence of the coexistence of short/long sleep duration and depression with regard to the CVD risk to date among middle-aged and elderly people.

Herein, in this study, we attempted to investigate the association between short/long sleep duration, depression and the risk of CVD based on the China Health and Retirement Longitudinal Study (CHARLS) database, and evaluate a joint effect of short/long sleep duration and depression on the CVD risk.

## Methods

### Data sources

All data in this retrospective cohort study were obtained from the CHARLS database [14], which is a nationally representative investigation of Chinese adults with 45 years or older. The investigation aimed at assessing the social, economic and health circumstances of residents. Respondents were followed every 2-3 years by conducting face-to-face computer-assisted personal interviews, physical measurements and blood tests. The baseline survey was carried out in 2011, with three follow-up surveys conducted in 2013, 2015, and 2018 [15, 16]. http://charls.pku.edu.cn/

### Study eligibility criteria

**Fig. 1:**
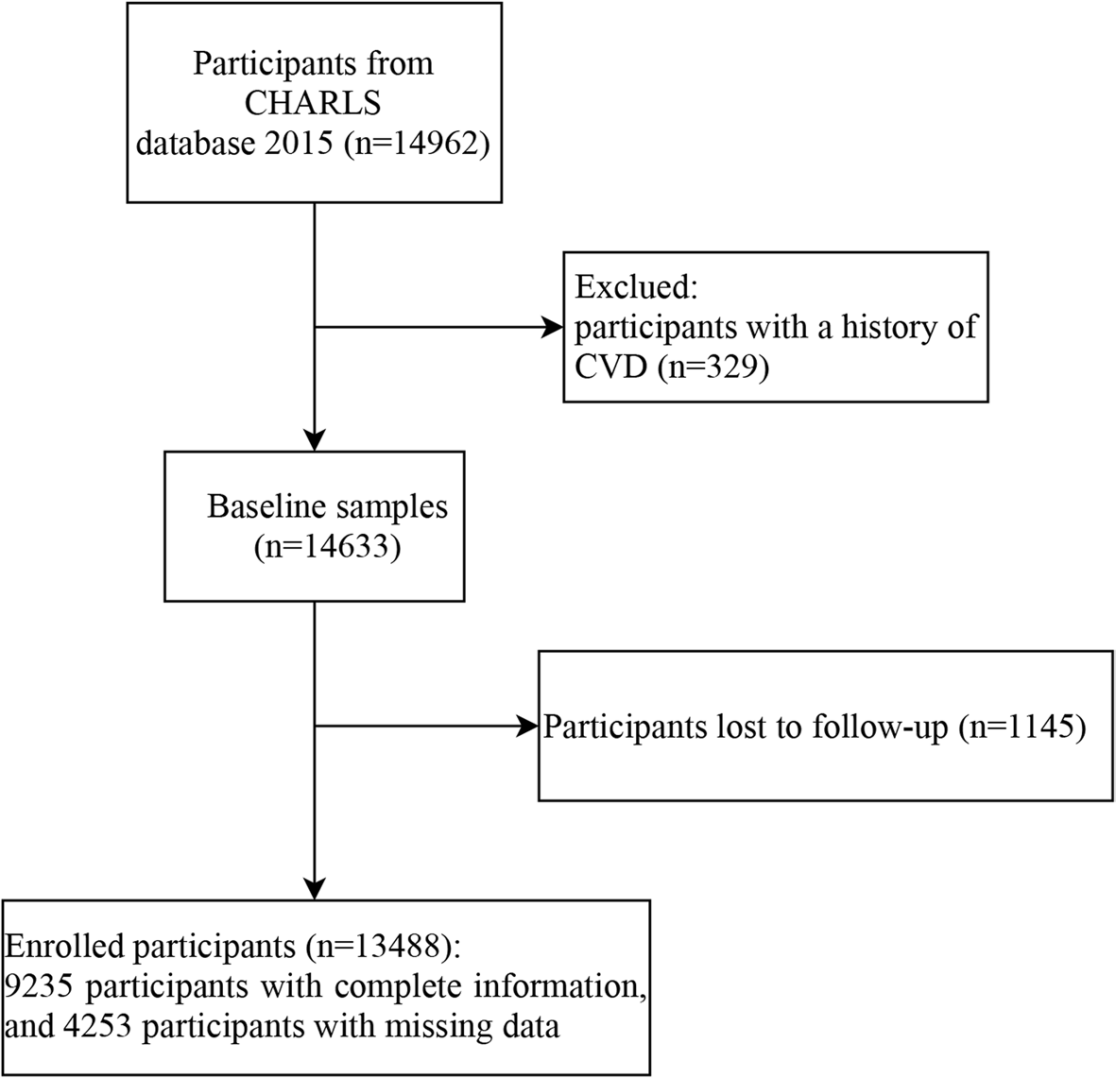
Flow chart of participants. 4253 participants had missing data and were treated with multiple imputation

Due to the high rate of lost follow-up for the included population of CHARLS database before 2015, in this retrospective cohort study, we chose the baseline data in the CHARLS database 2015, and follow-up data in 2018. Included criteria: participants had information about sleep duration and depression in the CHARLS database 2015 (*n*=14,962). Excluded criteria: participants already diagnosed with CVD before survey in 2015 (Fig. 1). All interviewees in CHARLS database needed to sign informed consent, and Biomedical Ethics Review Committee of Peking University approved the ethical review for the data collection in CHARLS database [14], thus according to the Ethics Review Committee of Guang’anmen Hospital, China Academy of Chinese Medical Sciences, secondary database analysis has been exempted from an ethical review.

### Data collection

Baseline variables and laboratory indicators were collected, including age (years), sex, educational background, marital status, deposit (CN¥), disability, exercise time (h/day), drinking, smoking, sleep time (h/day), depression, nap (min/day), chronic kidney disease (CKD), dyslipidemia, sleep disturbance, the use of hypnotics, diabetes, CVD, triglyceride (TG, mg/dl), high-density lipoprotein (HDL, mg/dl), low-density lipoprotein (LDL, mg/dl), systolic blood pressure (SBP, mmHg), diastolic blood pressure (DBP, mmHg), total cholesterol (TC, mg/dl), blood glucose (GLU, mg/dl), glycosylated hemoglobin (GHB, %).

Sleep duration was assessed by the respondents’ self-reported question which asked, “During the past month, how many hours of actual sleep did you get at night (average hours for one night)? This may be shorter than the number of hours you spend in bed.” The short, normal and long sleep duration were defined as <6 h, 6-8 h, >8 h, respectively [17]. Nap duration was measured by the following question “During the past month, how long did you take a nap after lunch on average?” (0 represent that respondent did not nap duration) [14]. Sleep disturbance was defined as how many days a week did participants have trouble falling asleep, frequently nighttime awakenings and earlier waking [18]: rarely or none of the time (<1 day), some or a little of the time (1-2 days), occasionally or a moderate amount of the time (3-4 days), and most or all of the time (5-7 days). The Epidemiological Studies Depression Scale (CES-D) was used to assess depression, which has been used to measure depression of the population [19]. The scale options consisted of 4 levels and were assigned: “rarely or none of the time=0”, “some or few times=1”, “occasionally or moderate number of times=2”, “most or all of the time=3”; The total score ranges from 0 to 30, with a scores ≥10 were defined as having depression [20]. Hypertension, CKD, dyslipidemia and diabetes was assessed by a self-report of physician's diagnosis: Have you been diagnosed with hypertension, CKD, dyslipidemia or diabetes by a doctor? Participants who answered “yes” to the question were defined as having hypertension, CKD, dyslipidemia or diabetes [21].

### Outcomes

Outcome variable was defined as the occurrence of CVD in the present study. The CVD was assessed by the following questions: “Have you been told by a doctor that you have been diagnosed with a stroke” or “Have you been diagnosed with heart attack, coronary heart disease, angina, congestive heart failure, or other heart problems?” Participants who answered “yes” to the question during the follow-up period were defined as having CVD [22].

### Statistical analysis

The normality test of measurement data was conducted by Shapiro-Wilk, normal distribution data was exhibited as mean ± standard deviation (Mean ± SD), and comparison between groups adopted independent sample t-test and ANOVA was used for comparison between multiple groups. Non-normal data were described in terms of

median and interquartile range [M (Q1, Q3)], and the comparison between groups was performed by Mann-Whitney U test and Kruskal-Wallis H test was used for comparison between multiple groups. The enumeration data were expressed as number of cases and composition ratio n (%), Chi-square or Fisher's exact test was used for comparison between two groups.

We adopted the univariate negative binomial regression model to explore the possible covariates that were associated with CVD. Then, multivariate negative binomial regression model was carried out to assess the statistical correlation of sleep duration and depression on CVD separately. Three models were used in this study. Model 1 was regarded as unadjusted; Model 2 adjusted several covariates that were performed for statistically significant in univariate analysis and had an impact on CVD in the literature, including age, sex, educational background, marital status, exercise time, chronic kidney disease, hypertension, diabetes, dyslipidemia, the use of hypnotics, disability, nap, drinking and deposit; Model 3 adjusted age, sex, educational background, marital status, exercise time, chronic kidney disease, hypertension, diabetes, dyslipidemia, the use of hypnotics, disability, nap, drinking, deposit, sleep disturbance, HDL, TC, TG, GLU and GHB. Additionally, we used the multivariate negative binomial regression models to evaluate the joint effect of sleep duration and depression on the CVD risk in different populations. Relative risk (RR) with 95% confidence interval (CI) was reported. With respect to missing data of the variables, we adopted multiple interpolation method. The data were interpolated for five times, and five datasets were generated. In the five datasets, the mean of the data with five times interpolations was taken for measurement data, and the mode of the data interpolated for five times was taken for enumeration data. A new interpolated dataset was obtained for subsequent analysis. Sensitivity analysis of missing data before and after interpolation was shown in Supplemental Table 1. Smoking data was missing too much and not participated in the analysis. We used the SAS (version 9.4, SAS institute., Cary, NC, USA) software for the statistical analysis and R (version 4. 0. 3, Mice package) for the multiple interpolation. Statistical tests were performed by using bilateral tests. *P*<0.05 was regarded as statistically significant.

## Results

### Baseline characteristics

After excluded some participants who were diagnosed with CVD before survey (*n*=329), and we also excluded 1,145 participants did not record whether CVD occurred at the end of the follow-up. A total of 13,488 eligible participants were enrolled in this retrospective cohort study eventually, with an average follow-up time of 2.7 years. There are 1,563 (11.59%) incident cases of CVD identified at the follow-up period to 2018. All participants’ characteristics were shown in Table 1. The study subjects’ average age was 57.89 ± 9.87 years. Furthermore, 3,772 (27.97%) participants had short sleep duration and 1,231 (9.13%) had long sleep duration, 4,091 (30.33%) had depression. It is worth noting that the population proportion of CVD occurrence among short sleep duration was higher than normal and long sleep duration groups, and the CVD occurred more frequently in the depression group than the non-depression group. Detailed baseline information was given in Table 1.

**Table 1:** Baseline characteristics of participants

Variables	Total (*n*=13488)	Sleep duration group			*P*	Depression group		*P*
		Short sleep duration (*n*=3772)	Normal sleep duration (*n*=8485)	Long sleep duration (*n*=1231)		Non-depression (*n*=9397)	Depression (*n*=4091)	
Age, years, Mean ± SD	57.89 ± 9.87	59.66 ± 9.94	56.91 ± 9.53	59.27 ± 10.96	<0.001	57.48 ± 9.84	58.84 ± 9.87	<0.001
Sex, n (%)					<0.001			<0.001
Male	6623 (49.10)	1632 (43.27)	4437 (52.29)	554 (45.00)		5042 (53.66)	1581 (38.65)	
Female	6865 (50.90)	2140 (56.73)	4048 (47.71)	677 (55.00)		4355 (46.34)	2510 (61.35)	
Education, n (%)					<0.001			<0.001
Illiterate	810 (6.01)	282 (7.48)	418 (4.93)	110 (8.94)		498 (5.30)	312 (7.63)	
Primary school	11616 (86.12)	3258 (86.37)	7313 (86.19)	1045 (84.89)		8055 (85.72)	3561 (87.04)	
Middle school	695 (5.15)	163 (4.32)	485 (5.72)	47 (3.82)		531 (5.65)	164 (4.01)	
High school or above	367 (2.72)	69 (1.83)	269 (3.17)	29 (2.36)		313 (3.33)	54 (1.32)	
Marital status, n (%)					<0.001			<0.001
Married	11360 (84.22)	3004 (79.64)	7345 (86.56)	1011 (82.13)		8117 (86.38)	3243 (79.27)	
Others	2128 (15.78)	768 (20.36)	1140 (13.44)	220 (17.87)		1280 (13.62)	848 (20.73)	
Deposit, n (%)					<0.001			<0.001
CN¥	9519 (70.57)	2829 (75.00)	5744 (67.70)	946 (76.85)		6262 (66.64)	3257 (79.61)	
≥2000 CN¥	3969 (29.43)	943 (25.00)	2741 (32.30)	285 (23.15)		3135 (33.36)	834 (20.39)	
Disability, (Yes), n (%)	347 (2.57)	127 (3.37)	190 (2.24)	30 (2.44)	0.001	184 (1.96)	163 (3.98)	<0.001
Exercise time, h/day, n (%)					0.005			<0.001
No exercise	8073 (59.85)	2256 (59.81)	5047 (59.48)	770 (62.55)		5630 (59.91)	2443 (59.72)	
hday	3274 (24.27)	887 (23.52)	2132 (25.13)	255 (20.71)		2355 (25.06)	919 (22.46)	
≥2 h/day	2141 (15.87)	629 (16.68)	1306 (15.39)	206 (16.73)		1412 (15.03)	729 (17.82)	
Drinking, n (%)	5133 (38.06)	1337 (35.45)	3394 (40.00)	402 (32.66)	<0.001	3818 (40.63)	1315 (32.14)	<0.001
Nap^a^, (min/day), M (Q1, Q3)	30.00 (0.00, 60.00)	1.00 (0.00, 60.00)	30.00 (0.00, 60.00)	30.00 (0.00, 90.00)	<0.001	30.00 (0.00, 60.00)	2.00 (0.00, 60.00)	<0.001
CKD, (Yes), n (%)	270 (2.00)	106 (2.81)	145 (1.71)	19 (1.54)	<0.001	149 (1.59)	121 (2.96)	<0.001
Diabetes, (Yes), n (%)	867 (6.43)	271 (7.18)	514 (6.06)	82 (6.66)	0.060	533 (5.67)	334 (8.16)	<0.001
Dyslipidemia, (Yes), n (%)	226 (1.68)	77 (2.04)	138 (1.63)	11 (0.89)	0.021	133 (1.42)	93 (2.27)	<0.001
Hypertension, (Yes), n (%)	3376 (25.03)	972 (25.77)	2035 (23.98)	369 (29.98)	<0.001	2347 (24.98)	1029 (25.15)	0.828
Sleep disturbance					<0.001			<0.001
Rarely or none of the time (<1 day)	7401 (54.87)	1092 (28.95)	5386 (63.48)	923 (74.98)		6444 (68.58)	957 (23.39)	
Some or a little of the time (1-2 days)	1885 (13.98)	510 (13.52)	1248 (14.71)	127 (10.32)		1286 (13.69)	599 (14.64)	
Occasionally or a moderate amount of the time (3-4 days)	1804 (13.37)	699 (18.53)	1025 (12.08)	80 (6.50)		787 (8.38)	1017 (24.86)	
Most or all of the time (5-7 days)	2398 (17.78)	1471 (39.00)	826 (9.73)	101 (8.20)		880 (9.36)	1518 (37.11)	
The use of hypnotics, (Yes), n (%)	10 (0.07)	4 (0.11)	6 (0.07)	0 (0.00)	0.317	2 (0.02)	8 (0.20)	0.002
SBP, mmHg, Mean ± SD	126.89 ± 19.27	127.53 ± 19.26	126.34 ± 19.01	128.70 ± 20.83	<0.001	127.02 ± 19.05	126.59 ± 19.75	0.246
DBP, mmHg, Mean ± SD	75.41 ± 11.17	75.10 ± 11.10	75.47 ± 11.15	75.95 ± 11.49	0.052	75.72 ± 11.17	74.70 ± 11.15	<0.001
TG, mg/dl, M (Q_1_, Q_3_)	119.47 (84.07, 182.30)	115.93 (82.30, 173.45)	119.47 (84.07, 185.84)	123.01 (84.96, 185.84)	0.004	119.47 (84.07, 184.07)	117.70 (84.07, 178.76)	0.243
HDL, mg/dl, Mean ± SD	50.93 ± 11.57	52.11 ± 11.78	50.54 ± 11.44	50.05 ± 11.54	<0.001	50.55 ± 11.35	51.80 ± 12.00	<0.001
LDL, mg/dl, Mean ± SD	100.80 ± 28.20	101.58 ± 27.67	100.55 ± 28.58	100.14 ± 27.12	0.120	100.98 ± 28.18	100.40 ± 28.25	0.275
TC, mg/dl, Mean ± SD	183.57 ± 37.44	184.95 ± 37.09	183.16 ± 37.92	182.11 ± 35.04	0.019	183.40 ± 37.07	183.96 ± 38.27	0.431
GLU, mg/dl, Mean ± SD	95.50 (88.29, 106.31)	95.50 (88.29, 104.50)	95.50 (88.29, 106.31)	95.50 (86.49, 106.31)	0.870	102.03 ± 31.11	102.61 ± 33.63	0.342
GHB, %, Mean ± SD	5.91 ± 0.92	5.93 ± 0.94	5.90 ± 0.90	5.93 ± 0.94	0.240	5.90 ± 0.87	5.95 ± 1.02	0.003
CVD, (Yes), n (%)	1563 (11.59)	517 (13.71)	891 (10.50)	155 (12.59)	<0.001	923 (9.82)	640 (15.64)	<0.001

*CVD* Cardiovascular disease, *TG* Triglyceride, *HDL* High-density lipoprotein, *LDL* Low-density lipoprotein, *SBP* Systolic blood pressure, *DBP* Diastolic blood pressure, *TC* Total cholesterol, *GLU* Blood glucose, *GHB* Glycosylated hemoglobin, *CKD* Chronic kidney disease, *others* separated, divorced, widowed, never married and cohabitated

^a^0 represent that respondent did not nap duration

### Effect of sleep duration/ depression on CVD

Some possible variables that were associated with CVD were shown in Table 2 (*P*<0.05) by univariate negative binomial regression model. The effects on CVD of sleep duration were presented in the Table 3. Model 1 (RR=1.03, 95%CI:1.02-1.04) showed that the risk of CVD in the short sleep duration group was increased in comparison with the normal sleep duration group, with similar results in Model 2 (RR=1.02, 95%CI:1.01-1.03) and Model 3 (RR=1.02, 95%CI:1.01-1.03). While the results of Model 1, Model 2 and Model 3 demonstrated that there was no significant difference between long sleep duration group and CVD (*P*>0.05).

**Table 2:** The possible variables that were associated with CVD

Variables	RR (95%CI)	*P*
Age, 10years	1.35 (1.30-1.41)	<0.001
Sex		
Male	Ref	
Female	0.99 (0.98-0.99)	0.026
Education background		
Illiterate	Ref	
Primary school	0.99 (0.96-1.04)	0.766
Middle school	0.97 (0.95-0.99)	0.028
High school or above	0.97 (0.94-1.01)	0.099
Marital status		
Married	Ref	
Others	1.01 (1.00-1.03)	0.098
Deposit		
<2000 CN¥	Ref	
≥2000 CN¥	0.98 (0.96-0.99)	0.002
Disability		
No	Ref	
Yes	1.07 (1.03-1.11)	<0.001
Exercise time		
No exercise	Ref	
hday	1.01 (0.99-1.02)	0.443
≥2 h/day	1.00 (0.98-1.01)	0.805
Drinking		
No	Ref	
Yes	0.98 (0.96-0.99)	<0.001
Nap	1.01 (1.01-1.01)	0.003
CKD		
No	Ref	
Yes	1.01 (0.99-1.01)	0.602
Diabetes		
No	Ref	
Yes	1.12 (1.09-1.14)	<0.001
Dyslipidemia		
No	Ref	
Yes	1.12 (1.08-1.17)	<0.001
Hypertension		
No	Ref	
Yes	1.08 (1.07-1.09)	<0.001
Sleep disturbance		
Rarely or none of the time (<1 day)	Ref	
Some or a little of the time (1-2 days)	1.01 (1.00-1.03)	0.153
Occasionally or a moderate amount of the time (3-4 days)	1.03 (1.01-1.05)	<0.001
Most or all of the time (5-7 days)	1.05 (1.03-1.07)	<0.001
The use of hypnotics		
No	Ref	
Yes	1.09 (0.89-1.33)	0.406
SBP, 10mmHg	1.17 (1.44-1.19)	<0.001
DBP, 10mmHg	1.18 (1.13-1.23)	<0.001
TC, 10 mg/dl	1.01 (0.99-1.02)	0.265
TG, 10 mg/dl	1.01 (0.99-1.01)	0.267
HDL, 10 mg/dl	0.94 (0.90-0.98)	0.003
LDL, 10 mg/dl	1.01 (0.99-1.03)	0.095
GLU, 10 mg/dl	1.02 (1.01-1.04)	<0.001
GHB, %	1.16 (1.12-1.19)	<0.001

*CVD* Cardiovascular disease, *TG* Triglyceride, *HDL* High-density lipoprotein, *LDL* Low-density lipoprotein, *SBP* Systolic blood pressure, *DBP* Diastolic blood pressure, *TC* Total cholesterol, *GLU* Blood glucose, *GHB* Glycosylated hemoglobin, *CKD* Chronic kidney disease, *others* separated, divorced, widowed, never married and cohabitated.

**Table 3:** The effect of sleep duration and depression on the risk of CVD

Variables	Model 1		Model 2		Model 3	
	RR (95%CI)	*P*	RR (95%CI)	*P*	RR (95%CI)	*P*
Sleep duration						
<6 h/day	1.03 (1.02-1.04)	<0.001	1.02 (1.01-1.03)	0.001	1.02 (1.01-1.03)	0.001
6-8 h/day	Ref					
>8 h/day	1.02 (1.01-1.04)	0.032	1.01 (0.99-1.03)	0.452	1.01 (0.99-1.03)	0.457
Depression						
No	Ref					
Yes	1.06 (1.05-1.07)	<0.001	1.05 (1.04-1.06)	<0.001	1.05 (1.04-1.06)	<0.001

*CVD* Cardiovascular disease, *RR* Relative risk, *CI* Confidence interval

Model 1: unadjusted

Model 2: adjusted age, sex, educational background, marital status, exercise time, chronic kidney disease, hypertension, diabetes, dyslipidemia, the use of hypnotics, disability, nap, drinking and deposit

Model 3: adjusted age, sex, educational background, marital status, exercise time, chronic kidney disease, hypertension, diabetes, dyslipidemia, the use of hypnotics, disability, nap, drinking, deposit, high-density lipoprotein, triglyceride, total cholesterol, blood glucose and glycosylated hemoglobin

As presented in Table 3, the results of three models indicated the effects of depression on the risk of CVD. Compared with non-depression group, depression group had a 0.06-fold (Model 1: RR=1.06, 95%CI:1.05-1.07), 0.05-fold (Model 2: RR=1.05, 95%CI:1.04-1.06), and 0.05-fold (Model 3: RR=1.05, 95%CI:1.04-1.06) increased risk of CVD.

### The interaction between short sleep duration and depression on CVD in different populations

After incorporating short sleep duration, depression and the interaction term of short sleep duration and depression into multifactor negative binomial regression model, we found that the interaction between short sleep duration and depression on CVD was statistically significant in these patients with diabetes and was a multiplicative interaction (*P*<0.05, Table 4). However, with respect to the relationship between short sleep duration and depression on CVD for total population or hypertension population, no an interaction was observed (*P*>0.05, Table 4).

**Table 4:** The interaction between short sleep duration and depression on the risk of CVD

Variables	Model 1	Model 2	Model 3
	*P*	*P*	*P*
**Total population**			
Short sleep duration & depression	0.882	0.866	0.867
**Hypertension population**			
Short sleep duration & depression	0.725	0.885	0.908
**Non-hypertension population**			
Short sleep duration & depression	0.897	0.857	0.878
**Diabetes population**			
Short sleep duration & depression	0.048	0.047	0.042
**Non-diabetes population**			
Short sleep duration & depression	0.599	0.692	0.663

*CVD* Cardiovascular disease

Model 1: unadjusted

Model 2: adjusted age, sex, educational background, marital status, exercise time, chronic kidney disease, hypertension, diabetes, dyslipidemia, the use of hypnotics, disability, nap, drinking and deposit

Model 3: adjusted age, sex, educational background, marital status, exercise time, chronic kidney disease, hypertension, diabetes, dyslipidemia, the use of hypnotics, disability, nap, drinking, deposit, high-density lipoprotein, triglyceride, total cholesterol, blood glucose and glycosylated hemoglobin

## Discussion

In this analysis of 13,488 participants from CHARLS database, we revealed that short sleep duration and depression were independent risk factors for CVD occurrence; Importantly, we found that there might be an interaction between short sleep duration and depression in relation to an increased risk of CVD among middle-aged and elderly patients with diabetes.

For the present study, after adjusted covariates, the risk of CVD in people with independent short sleep duration and depression was 0.02 times and 0.05 times than those with normal sleep duration and without depression, respectively. There was no doubt that our result showed that short sleep duration and depression were associated with the increased risk of CVD, which were mostly in line with prior researches [9, 23–25]. Short sleep duration was associated with an increased levels of markers of inflammation, [26]. When short sleep duration triggered mild inflammation, leading to an increased stress response in the hypothalamic-pituitary-adrenal axis, which may cause the rise of blood pressure and an increased risk of CVD [26]. Not only that, short sleep duration could induce biological effects including the changes in neural autonomic control and coagulation responses, an elevated level of oxidative stress, and accelerated atherosclerosis, triggering metabolic disorders to raise the risk of CVD [25]. Likewise, the associated mechanisms of depression and CVD might be related to endothelial dysfunction, autonomic nerve dysfunction, inflammation and life behavior [27, 28]. To our knowledge, some studies also reported a link between long sleep duration and increased risk of CVD among elderly people, which might be associated with arterial stiffness, blood pressure variability, gluco-regulatory function and systemic inflammation [29, 30]. However, our study showed that there was no statistically difference between long sleep duration group and CVD risk among Chinese middle-aged and elderly individuals [31], and the reason may be due to the difference of sample size. Simultaneously, we also found that there were 9.13% Chinese middle-aged and elderly individuals with long sleep duration, and more studies are still warranted on this relationship of long sleep duration and CVD risk in the future.

For the present study, the interaction between short sleep duration and depression on CVD risk for total population was not observed. However, we found that the interaction between short sleep duration and depression might be associated with an increased risk of CVD for middle-aged and elderly patients with diabetes. In other words, when patients with diabetes suffered from both of symptom of short sleep duration and depression, there was a higher risk of CVD. Nowadays, diabetes has been considered as one of the most common chronic conditions, and its prevalence are increasing worldwide [32]. Previous studies have suggested that people with diabetes appear to be at greater risk of depression [32–34]. In addition, some studies have shown that people with both diabetes and depression could suffer poorer outcomes, such as poorer quality of life, poorer self-management of diabetes and poorer medical outcomes [35, 36], which also suggested an importance of paying attention to the prognosis of patients with diabetes and depression. Additionally, insufficient sleep duration was also highly prevalent in patients with diabetes [37], which may contribute to a poor prognosis in those patients. In our study, coexisting short sleep duration and depression may increase the risk of CVD in middle-aged and elderly patients with diabetes. Accordingly, this finding may support the viewpoint that, patients with diabetes should pay attention to their sleep duration and the occurrence of depression. Appropriate increase of sleep duration and physical activities, a healthy diet and psychological treatment may beneficial to prevent the risk of CVD in middle-aged and elderly patients with diabetes [38, 39]. Although we found an interaction between depression and sleep duration on the risk of CVD in middle-aged and elderly patients with diabetes, more prospective studies are needed in the future to validate our results and explore the possible mechanism.

The strengths of our study included a large sample size, make the findings more convincing; and the results also may provide a reference that with respect to patients with diabetes, they should pay more attention to both the sleep time and the occurrence of depression, to decrease the risk of CVD. There are limitations that cannot be ignored. Firstly, the issue whether eligible patients diagnosed as short/long sleep duration or depression have been treated not considered in the present study, which may not be got from CHARLS database. Secondly, the coexistence duration of short sleep duration and depression may influence risk of CVD, there was no information collected from CHARLS database about the duration of both short sleep duration and depression, and more trials still are needed to confirm this association. Thirdly, CVD was defined as outcome variable, contained heart attack, coronary heart disease, angina pectoris, congestive heart failure or other heart problems and stroke, but we don’t know is that the synergistic interaction between short sleep duration and depression increase the higher risk for what kind of diseases. Fourthly, smoking has long been considered an important risk factor for CVD [40]. But, in this study, the variable (smoking) was missing so much that we excluded it. This is a limitation of our study. Lastly, short observational period for observation the incidence of CVD needs to be noted in this study, and more prospective studies with long follow-up periods need to be conducted in the future to verify our results.

## Conclusion

In short, we found an interaction between depression and short sleep duration on the risk of CVD among middle-aged and elderly patients with diabetes. Patients with diabetes should pay more rigorous attention to their sleep duration and the occurrence of depression, and coexisting short sleep duration and depression may expose them to a higher risk of CVD. However, more researches are needed to confirm this association in the future.

## Supplementary Information

**Additional file 1:** **Supplemental Table 1.** Sensitivity analysis of missing data before and after interpolation.

## Data Availability

The datasets generated and/or analyzed during the current study are available in the CHARLS repository, http://charls.pku.edu.cn/.

## Abbreviations

CVD: Cardiovascular disease
CHARLS: China Health and Retirement Longitudinal Study
TG: Triglyceride
HDL: High-density lipoprotein
LDL: Low-density lipoprotein
SBP: Systolic blood pressure
DBP: Diastolic blood pressure
TC: Total cholesterol
GLU: Glucose
GHB: Glycosylated hemoglobin
CES-D: Epidemiological Studies Depression Scale
Mean ± SD: Mean ± standard deviation
M (Q1, Q3): Median and interquartile range
