# Supplementary material for: Interaction of sleep duration and depression on cardiovascular disease: a retrospective cohort study
Source: BMC Public Health. 2022 Sep 15;22:1752. doi: 10.1186/s12889-022-14143-3 (PMC9479441; doi:10.1186/s12889-022-14143-3)
Supplement: Supplementary file 1 — Additional file 1: Supplemental Table 1. Sensitivity analysis of missing data before and after interpolation. [file 12889_2022_14143_MOESM1_ESM.docx]

**Supplemental Table 1 Sensitivity analysis of missing data before and after interpolation**

| Variables | Lack of proportion (%) | Before interpolation | After interpolation | Statistics | *P* |
| --- | --- | --- | --- | --- | --- |
| Age | 1.03 | 57.89 ± 9.87 | 57.89 ± 9.87 | t=0.03 | 0.975 |
| Deposit | 4.57 |  |  | χ^2^=0.006 | 0.940 |
| <2000 |  | 9089 (70.62) | 9519 (70.57) |  |  |
| ≥2000 |  | 3782 (29.38) | 3969 (29.43) |  |  |
| Disability | 4.25 |  | 273 (2.53) | χ^2^=0.001 | 0.977 |
| No |  | 12582 (97.42) | 13141 (97.43) |  |  |
| Yes |  | 333 (2.58) | 347 (2.57) |  |  |
| Drinking | 0.04 |  |  | χ^2^=0.000 | 0.997 |
| No |  | 8351 (61.94) | 8355 (61.94) |  |  |
| Yes |  | 5131 (38.06) | 5133 (38.06) |  |  |
| Hypertension | 17.70 |  |  | χ^2^=0.330 | 0.566 |
| No |  | 8287 (74.65) | 10112 (74.97) |  |  |
| Yes |  | 2814 (25.35) | 3376 (25.03) |  |  |
| Nap | 0.28 | 30.00 (0.00, 60.00) | 30.00 (0.00, 60.00) | Z=0.034 | 0.973 |
| SBP | 17.70 | 127.06 ± 19.31 | 126.89 ± 19.27 | t=-0.70 | 0.481 |
| DBP | 17.70 | 75.27 ± 11.17 | 75.41 ± 11.17 | t=0.98 | 0.328 |
| TC | 31.52 | 183.43 ± 36.33 | 183.57 ± 37.44 | t=0.28 | 0.780 |
| TG | 31.52 | 112.39 (81.42, 167.26) | 119.47 (84.07, 182.30) | Z=-7.034 | <0.001 |
| HDL | 31.52 | 51.51 ± 11.45 | 50.93 ± 11.57 | t=-3.72 | <0.001 |
| LDL | 31.53 | 102.03 ± 28.67 | 100.80 ± 28.20 | t=-3.21 | 0.001 |
| GLU | 31.53 | 102.28 ± 32.69 | 102.20 ± 31.90 | t=-0.17 | 0.867 |
| GHB | 31.25 | 5.93 ± 0.94 | 5.91 ± 0.92 | t=-1.07 | 0.287 |

Note: CVD: cardiovascular disease; TG: triglyceride; HDL: high-density lipoprotein; LDL: low-density lipoprotein; SBP: systolic blood pressure; DBP: diastolic blood pressure; TC: total cholesterol; GLU: glucose; GHB: glycosylated hemoglobin. The process of multiple imputation as follows: The data were interpolated for five times, and five datasets were generated. In the five datasets, the mean of the data with five times interpolations was taken for measurement data, and the mode of the data interpolated for five times was taken for enumeration data. A new interpolated dataset was obtained for subsequent analysis. “After interpolation” means “a new interpolated dataset”.
